# Supplementary material for: Identification of ubiquitin Ser57 kinases regulating the oxidative stress response in yeast
Source: eLife. 2020 Oct 19;9:e58155. doi: 10.7554/eLife.58155 (PMC7647399; doi:10.7554/eLife.58155)
Supplement: Supplementary file 1. — Yeast cells (NHY318 background) expressing either wildtype or S57D ubiquitin were cultured in heavy (H; expressing wildtype ubiquitin) or light (L, expressing S57D ubiquitin) SILAC media to the mid-log phase and treated with 1 mM H2O2 for 30 min before harvesting cells. Following cell lysis and digestion of lysates with trypsin for 24 hr, ubiquitin-remnant peptides were enriched (see Materials and methods) and analyzed by mass spectrometry. Three biological replicate experiments were analyzed. Since the peptide corresponding to K63-linked poly-ubiquitin also harbors the residue mutated in phosphomimetic (S57D) ubiquitin, K63 linkages are a blind spot for SILAC measurements in these experiments. ‘n.d.’ indicates not detected. [file elife-58155-supp1.docx]

**Supplementary File 1**

| **Peptide** | **Linkage type** | **L:H SILAC Ratio** | | | | |
| --- | --- | --- | --- | --- | --- | --- |
|  |  | ***Rep 1*** | ***Rep 2*** | ***Rep 3*** | **Mean** | **St. Dev.** |
| MQIFVK**[GG]**TLTGK | K6 | n.d. | 1.20 | 1.31 | 1.26 | 0.08 |
| IQDK**[GG]**EGIPPDQQR | K33 | 0.90 | 0.90 | 0.92 | 0.91 | 0.01 |
| LIFAGK**[GG]**QLEDGR | K48 | 1.29 | 1.43 | 1.43 | 1.39 | 0.08 |
