# Supplementary material for: Identification of ubiquitin Ser57 kinases regulating the oxidative stress response in yeast
Source: eLife. 2020 Oct 19;9:e58155. doi: 10.7554/eLife.58155 (PMC7647399; doi:10.7554/eLife.58155)
Supplement: Supplementary file 2. — For yeast Ser57 ubiquitin kinases, we analyzed consensus phosphorylation motifs as determined from a previous study based on in vitro activity analysis on peptide libraries (Mok et al., 2010). Values in parentheses are the quantified selectivity values, based on site preference of in vitro activity. Only amino acids selected at a position with a value >2 are shown. [file elife-58155-supp2.docx]

**Supplementary File 2**

|  | **position** | | | | | | | | | |
| --- | --- | --- | --- | --- | --- | --- | --- | --- | --- | --- |
|  | -5 | -4 | -3 | -2 | -1 | 0 | +1 | +2 | +3 | +4 |
| **ubiquitin** | D | G | R | T | L | S | D | Y | N | I |
| Vhs1 | M (6.4) | R (2.1) | R (11.1) | S (3.1)  T (2.7)  R (2.0) | -- | S (11.1)  T (8.9) | -- | S (2.7)  T (2.0) | -- | -- |
| Gin4 | L (2.0) | R (2.7)  K (2.4) | R (9.9) | S (7.9) | M (2.9) | S (14.5)  T (7.5) | M (2.6) | W (2.4)  Y (2.2) | N (2.8) | L (3.9)  I (2.3) |
| Kcc4 | Y (2.0) | R (2.4)  K (2.3) | R (7.6) | S (3.9)  T (2.1) | -- | S (17)  T (5) | -- | C (2.7)  I (2.6) | N (2.4) | L (2.4) |
